# Dengue infections in India: A meta-analysis

**DOI:** 10.6026/9732063002001221

**Published:** 2024-10-31

**Authors:** Dhirajkumar Mane, Satish V. Kakade, Supriya S. Patil

**Affiliations:** 1 Directorate of Research, Krishna Vishwa Vidyapeeth (Deemed to Be) University, Karad, Maharashtra, India; 2 Department of Community Medicine, Krishna Vishwa Vidyapeeth (Deemed to Be) University, Karad, Maharashtra, India

**Keywords:** Dengue, dengue virus, burden of dengue, sero-prevalence, prevalence

## Abstract

The escalating impact of dengue infection on health and mortality is a critical global issue. Therefore, it is of interest to assess the current trends of dengue infection in India. We searched through a wide range of internet databases to gather comprehensive studies on the incidence, prevalence, sero-prevalence, cost effectiveness and mortality rate of dengue infection in India from 2014 to 2023 (10 years) in a total of 127 studies. Analysis shows significant heterogeneity (diversity) in reported outcomes (p-values < 0.001). Thus, public health strategies should include early detection of dengue infection in our country.

## Background:

Dengue is caused by an arbovirus of the Genus Flavivirus and Family Flaviviridae, is one of the most prevalent, fast-spreading vector-borne diseases impacting people [1]. As a result, research has shown that dengue disease may be clinically characterized as either mild dengue, dengue with or without warning signals, or severe dengue [1, 2]. According to a study, an estimated 105 million infections occur worldwide every year, only 51 million of which are symptomatic, making it a major public health issue [3]. Due to increasing worldwide travel and the geographical expansion of the Aedes vector mosquitoes, dengue virus are transmitted on all major continents, with new cases occurring and spreading to formerly non-endemic locations [4]. The primary dengue infection is presumed to provide permanent sterilizing immunity against homologous serotypes; however, exceptions exist in human and animal experimental investigations [5, 6]. Secondary infection (SC) with an un-encountered serotype often leads to classical dengue fever (fever) and is linked to a heightened risk of severe sequelae [7, 8]. This is a significant risk factor for the heightened severity of dengue fever via the antibody-dependent enhancement (ADE) pathway [9]. A second dengue fever occurring within two years after the first infection is likely to be an asymptomatic infection, as shown by the neutralizing antibody titer [10]. Therefore, it is of interest to assess dengue fever in India with the help of systematic review (SR) and meta-analysis (MA).

## Material and Method:

## Protocol development:

In the present manuscript, written according to the PRISMA checklist, [11] only the scientific evidence of dengue infection current Trent in India was investigated. This SR protocol was a priori registered in The International Prospective Register of SR (Registration No: CRD42024552341).

## Search strategy, Databases & Selection criteria:

We have searched in electronic databases such as Cochrane Library, Medline, Web of Science (WoS), PubMed, Scopus & Google Scholar for publications published between January 2014 and December 2023. Appendix I: Search Strategy contains all of the search strategy's details. We have specifically used date/year as a filter to search three databases i.e. (PubMed, Scopus/Elsevier and Embase) from May 24-27, 2024. The Covidence application was used to screen abstracts.

## Inclusion criteria:

[1] All studies conducted in India on this topic regardless of their design, purpose or population.

[2] Incidence

[3] Prevalence

[4] Number of cases

[5] Mortality

[6] Burden

[7] Complications

[8] Virus serotype details / seroprevalence

2 reviewers independently collected data from selected papers using a predefined data extraction form. Any discrepancies in it were resolved through consensus. The information that was extracted from studies includes year of publication, study setting, location, period, laboratory investigations, number of suspected patients tested & found positive, the age distribution of cases and details of dengue serotypes as shown in Table 1, Table 2, Table 3, Table 4, Table 5 to Table 6 (dataset I -VI).

## Data extraction & Review synthesis:

3 reviewers carried out the initial screening. The collected literature was first searched to remove duplicates before being entered into Rayyan software [132]. After that, the titles and abstracts were screened. In 2nd screening phase, 3 reviewers evaluated the selected papers based on their compliance with the eligibility standards. While the 2, independently shortlisted studies that met the design, participant and result requirements. Disagreements were resolved by discussion and, if necessary, the involvement of a 3rd reviewer. Using a pre-designed data extraction form in Microsoft Excel, 3 reviewers independently gathered details from the selected research. Initially, the search results were imported into Mendeley software (Version 1.19.6) where duplicate records were removed.

## The outcome measures were:

[1] The prevalence of laboratory-confirmed dengue infection among clinically suspected patients in the research area, as reported in hospital/laboratory or community-based investigations during outbreaks.

[2] Seroprevalence of dengue in the study population dengue fever conditions, dengue severity and Mortality rate among dengue patients those were confirmed in labs.

[3] Primary and secondary infections present.

[4] Cost of illness/burden which included reported direct and indirect costs associated with dengue hospitalization.

[5] The non-structural protein-1 (NS1) antigen, immunoglobulin M (IgM) antibodies against dengue virus, haem-agglutination inhibition (HI) antibodies against dengue virus, RT-PCR positivity, or virus isolation was used to diagnose acute dengue infection in the clinically suspected patients. The measurement of IgG or neutralizing antibodies against the dengue virus was used to determine the seroprevalence of dengue.

## Quality/Risk of bias assessment:

We utilized a modified version of the Joanna Briggs Institute (JBI) appraisal checklist for assessing prevalence data [133], along with key components from the Strengthening the Reporting of Observational Studies in Epidemiology (STROBE) checklist [134] to gauge potential bias. Our primary criteria for bias assessment included outcome variables, laboratory testing procedures and participant selection strategies (refer to Supplementary file S2 Appendix). 2nd reviewers independently evaluated bias risk, resolving any disagreements through discussion. In cases of unresolved disputes, the perspective of a 3rd reviewer was sought and any disagreements were resolved. When needed, the viewpoint of the 3rd reviewer was sought.

## Statistical analysis:

Using the single user licenced version of STATA 18.5 StataCorp LLC, Texas, USA, software and R-Studio analysis was carried out. The proportions from the combined data were shown along with their 95% confidence intervals (CI). Heterogeneity was assessed using an I2-test, where values below 25% indicated mild heterogeneity, values between 25 and 75% indicated moderate heterogeneity and values over 75% indicated significant heterogeneity [15, 16]. Based on the inverse variance approach for weighting, the Der-Simonian-Laird method for a random-effects model was used to compute the total pooled prevalence. Both the pooled estimates for the general and subgroup analyses and the study-specific estimates for each participant were shown using forest plots. To further demonstrate publication bias, a funnel plot was made.

## Results:

Initially, we searched 6582 published articles in various electronic databases such as PubMed-2281, Ovid/Medline-47, Web of Science-4037 and Google Scholar-217 published. This was later on narrowed down to 999 unique articles after duplicate removal over the last 10 years. Following titles and abstracts screening, 613 articles were excluded, leaving 386 articles for full-text evaluation. This resulted in 127 studies being selected for analysis [17, 18, 19, 20, 21, 22, 23, 24, 25, 26, 27, 28, 29, 30, 31, 32, 33, 34, 35, 36, 37, 38, 39, 40, 41, 42, 43, 44, 45, 46, 47, 48, 49, 50, 51, 52, 53, 54, 55, 56, 57, 58, 59, 60, 61, 62, 63, 64, 65, 66, 67, 68, 69, 70, 71, 72, 73, 74, 75, 76, 77, 78, 79, 80, 81, 82, 83, 84, 85, 86, 87, 88, 89, 90, 91, 92, 93, 94, 95, 96, 97, 98, 99, 100, 101, 102, 103, 104, 105, 106, 107, 108, 109, 110, 111, 112, 113, 114, 115, 116, 117, 118, 119, 120, 121, 122, 123, 124, 125, 126, 127, 128, 129, 130, 131, 132, 133, 134, 135, 136, 137, 138, 139-140] as shown in (Figure 1 see PDF).

## Prevalence/proportion of laboratory dg cases & outbreak:

The clinically suspected patients are provided by 78 out of the 127 published studies included in this synthesis. This comprised 8 studies reporting outbreak investigations and 71 studies conducted in hospital or laboratory settings. A proportion of the studies that the hospital validated were conducted at the time; that the affected areas were going through an outbreak. The data of laboratory-confirmed cases by month were supplied by 32 research (40.5%) out of the 79 studies that reported a proportion of dengue cases; the majority of these studies (n = 53, 67%) indicated increased dengue positivity throughout the rainy seasons, particularly from July to October. The majority of the forty-seven investigations identified acute dengue infection using a single test, as follows: detection of the NS1 antigen = 1, virus isolation = 1, RT-PCR (Real-Time Reverse Transcription - Polymerase Chain) = 7, haem-agglutination inhibition antibodies = 2 and IgM antibodies = 36. The other studies employed multiple tests.

## Case definitions used:

While discussed about case studies their; we took assistance of WHO (World Health Organizations), NVBDCP (National Center for Vector Borne Diseases Control) & AFI (Acute Febrile Illness) case definitions. Out of 79 studies during hospital settings majority n=53 were clinical suspected dengue followed by n=13 WHO case definition, n=9 AFI case definition and the remaining studies n=4 were used NVBDCP case definitions respectively. Both hospital confirmed dengue study and showed similarly, among 71 hospital confirmed dengue cases n=51 were clinical suspected dengue followed by n=9 WHO case definition, n=9 AFI case definition and the remaining studies n=2 were used NVBDCP case definitions respectively. Among the reported outbreaks, investigators used n=4 WHO case definition, n=2 AFI case definition and the remaining studies n=2 were used NVBDCP case definitions respectively.

## Dengue proportion in India:

Based on testing of 206783 clinically suspected individuals from 78 studies, the overall estimate of the prevalence of laboratory-confirmed dengue infection in the random effects model was 39.4% (95% CI: 35.6%-44.67%) as shown in (Figure 2 see PDF). The heterogeneity was assessed by Hedge g statistics. The heterogeneity overcomes by using random effect model as shown in (Figure 3 see PDF). The publication biased(PB) was assessed by using funnel plot, some asymmetry observed because individual study had different proportion and this was directly impacts on shifting the points on funnel to outside but the both the side almost normality hence in our study there was no publication bias was reporting as shown in. The prevalence reported by the 79 studies showed significant heterogeneity (LRT p<0.001). In comparison to hospital-based surveillance (HBS) studies (40%, 95% CI: 35-44), the prevalence of laboratory-confirmed dengue infection was nearly identical in studies reporting outbreaks (OB) or hospital-based surveillance studies during outbreaks (39%, 95% CI: 34-44).

## Age distribution:

Data was available for 30 out of 127 studies on laboratoryconfirmed DG cases. The overall average age of confirmed DG patients in this study was 24.47 years; with a standard deviation of 9.22 years with age range was 7 to 36 years as shown in (Figure 4 - see PDF).

## Dg-FV & Dg-S proportion:

31 studies provided information on dengue fever, while 32 studies provided information on dengue symptoms. The majority of the research (n = 19, 59.38%) utilized the WHO 1997 classification, while the remaining studies (n = 3, 9.38%) employed the WHO 2007 classification. Additionally, for dengue fever condition and severity, (n = 6, 18.75%) used the WHO 2009 classification, whereas 4 studies (12.5%) used the WHO 2012 classification. It was reported that between 31% and 100% of laboratory-confirmed patients had dengue fever. According to the random effect model, 75% (95% CI: 67-82) of laboratory-confirmed studies exhibited dengue fever overall. The Hedges g-Method (HD-M) was used to estimate the random effect model, indicating no heterogeneity as shown in (Figure 5 see PDF). Bias in publications observed and depicted that higher prevalence publications were more side. On the other hand, among patients with laboratory-confirmed, the reported percentage of dengue symptoms cases varied from 2% to 69%. In the random effect model (REM), the total percentage of dengue symptoms across laboratory-confirmed studies was 25% (95% CI: 19-31). The data on dengue symptoms showed no evidence of heterogeneity as shown in (Figure 6 see PDF).

## DG Mortality (MT) in India:

In the provided research, 48 provided information on MT rate of DG, It was reported that between 0% and 9% of LB-CN patients had DG-FV. According to the REM, 1% (95% CI: 1-2) of LB-CN studies exhibited DG-FV overall. The HD-M was used to estimate the REM, indicating no HTG. Bias in publications observed and depicted that higher prevalence publications was more side, The removal of the study with greatest weight in each LB-CN test of DG disease did not change the results.

## Pm-if & SC among dg cases in India:

A comprehensive analysis of 15 studies [31, 37, 48, 59-60, 71, 78, 81- 82, 89, 104- 105, 115, 124] enabled the categorization of LB-CN-DG-IF into PM and SC. The prevalence of initial DG-IF varied widely ranges from 32% to 97% across the studies. Overall, PM-DG-IF accounted for 77% of LB-CN cases (95% CI: 65-87). Meanwhile, SC-DG-IF occurred in 23% of LB-CN cases (95% CI: 13-35), with a range of 3% to 68% across the studies.

## PB-BA & sensitivity statistics (SS-ST):

There was no indication of publication bias in the dengue prevalence estimates from hospital-based studies with LB-CN cases, outbreaks & SP according to analysis utilizing funnel plots and the HD approach. The estimates of dengue severity and fatality did, however, reveal a substantial publication BA, with publications demonstrating higher prevalence being more likely to be published. However, sensitivity analysis showed that the pooled percentages of research results held steady, suggesting the estimates' resilience. The removal of the study with greatest weight in each dengue cases LB-CN did not change the results.

## Discussion:

The analysis primarily drew on data from HB and laboratorybased surveillance studies, as well as reports from investigations into dengue outbreaks. There have been more than 10 million reported cases of dengue along with over 5,000 dengue-related deaths across 80 countries. The Pan American Health Organization (PAHO) region has reported the majority of cases, with over nine million cases. Within the PAHO region, Brazil has reported the highest number of cases (over eight million), followed by Argentina, Paraguay, Peru and Colombia. In Europe, imported cases from endemic areas have been reported in Germany, Italy and France, but no locally acquired cases have been reported.

Dengue circulation has also been reported in the Southeast Asia and Western Pacific regions, as well as in Africa. It concentrated on their operations, implementation and structure. The WHO had set aggressive goals to cut dengue-related mortality by 50% and morbidity by 25% along with burden by 2020 [135-136]. A recent study in Brazil found a significant disparity in the infection rates between wealthy and disadvantaged youth. Specifically, the study revealed that 60% of young people from disadvantaged backgrounds were infected, which is three times the rate of their wealthier peers and our study also found similar kind of results where average age was 24.4 years [137]. Overall, 127 studies with a total of 3Lacs population were covered for study of dengue disease in our country. Viral assays are used in laboratories to confirm dengue infection (RNA detection by RTPCR, NS1 antigen detection by ELISA) [138]. The overall prevalence of dengue disease in our India based on testing of 206783 clinically suspected individuals from 79 studies, the overall estimate of the prevalence of laboratory-confirmed - dengue infection in the random effect model was 39.4% (95% CI: 35.6%-44.67%) According to a study, the overall prevalence of dengue in country like India based on testing 206783 clinically suspected individuals from 79 different studies was 39.4% [139].

There are also research gaps in India's understanding of dengue epidemiology and the fact that different types of the dengue virus are still being spread. These factors show that dengue is still a major public health issue in India. The high percentage of dengue-positive cases, severity and case mortality in India are all indicators that dengue continues to be a significant public health concern in the country. As a consequence of this, it is required to undertake community-based cohort studies that are wellstructured and cover a variety of geographical locations of the country in order to offer reliable estimates of the age-specific incidence of dengue fever in India [140].

## Conclusion:

DG-FV remains a pressing public health issue in India, as indicated by its high incidence, severity and mortality rates, as well as the circulation of multiple virus serotypes. To better comprehend the epidemic, we suggest conducting in-depth research, including community-based studies across various regions to determine age-specific incidence rates. Alternatively, a nationwide survey could be undertaken to determine age-specific seroprevalence rates, which also includes targeted studies in different geographic areas in India.

## Limitation:

[1] We have restricted our search to quantitative sides which might be neglected towards qualitative enrichment of variables

[2] We considered peer-reviewed journals database from certain articles, which lead to exclusion of government registries data as a grey literature that could provide other aspects of the picture too.

## Future research:

We should implement active surveillance systems, scaling up vector control measures, enhance more public awareness & education and finally, strengthen the prevention strategies.

## Figures and Tables

**Table 1:** Dataset I-DG Proportion

**Sr. No.**	**Reference No.**	**Author**	**Year of Publication**	**Year of study**	**Country**	**Study Type (Hospital/Outbreak)**	**Case Definition Referred**	**Number of patients tested (Total)**	**Number of people tested positive (Event)**
1	12	Abhilash *et al.*	2016	2012-2013	India	Hospital	AFI	1258	386
2	13	Afreen *et al.*	2015	20112014	India	Hospital	AFI	604	416
3	14	Ahir *et al.*	2016	2014-2015	India	Hospital	Clinical Suspected Dengue	1146	148
4	15	Ahmad *et al.*	2016	2012-2013	India	Hospital	AFI	298	93
5	16	Ahmed *et al.*	2015	2010	India	Hospital	Clinical Suspected Dengue	4370	1700
6	17	Amudhan *et al.*	2015	2010-2013	India	Hospital	Clinical Suspected Dengue	4578	1185
7	18	Anand *et al.*	2016	2011	India	Hospital	WHO	112	94
8	19	Arora et al.	2021	2015	India	Hospital	Clinical Suspected Dengue	647	170
9	20	Badoni *et al.*	2023	2018-2019	India	Hospital	Clinical Suspected Dengue	279	222
10	21	Barde *et al.*	2014	2011-2012	India	Hospital	NVBDCP	138	21
11	22	Barde *et al.*	2015	2013	India	Outbreak	NVBDCP	648	321
12	23	Barde *et al.*	2015	2012	India	Outbreak	WHO	247	115
13	24	Barua *et al.*	2016	2014	India	Hospital	AFI	156	101
14	25	Bhattacharya *et al.*	2017	2013	India	Hospital	Clinical Suspected Dengue	218	168
15	26	Biswas *et al.*	2014	2012	India	Outbreak	Clinical Suspected Dengue	100	79
16	27	Chakravarti *et al.*	2014	2013	India	Hospital	Clinical Suspected Dengue	700	280
17	28	Changal *et al.*	2016	2015	India	Hospital	Clinical Suspected Dengue	225	114
18	29	Deshkar *et al.*	2017	2012-2016	India	Hospital	Clinical Suspected Dengue	15606	3822
19	30	Dhingra *et al.*	2020	Feb 2014-Oct 2015	India	Hospital	Clinical Suspected Dengue	255	216
20	31	Dinkar *et al.*	2020	2012-2017	India	Hospital	Clinical Suspected Dengue	900	461
21	32	Duthade *et al.*	2015	2014	India	Hospital	Clinical Suspected Dengue	872	233
22	33	Gopal *et al.*	2016	2013	India	Hospital	Clinical Suspected Dengue	50	25
23	34	Gopinath *et al.*	2023	2018-2022	India	Hospital	Clinical Suspected Dengue	1383	286
24	35	Gusani *et al.*	2017	2014	India	Hospital	NVBDCP	765	331
25	36	Henna *et al.*	2014	2010-2012	India	Hospital	Clinical Suspected Dengue	7836	2807
26	36	Henna *et al.*	2014	2012-2013	India	Hospital	Clinical Suspected Dengue	2228	527
27	37	Islam *et al.*	2016	2015	India	Hospital	AFI	62	18
28	38	Jindal *et al.*	2014	2011	India	Hospital	Clinical Suspected Dengue	1787	586
29	39	Joshua *et al.*	2016	2014-2015	India	Hospital	Clinical Suspected Dengue	4952	2442
30	40	Kartick *et al.*	2017	2014	India	Outbreak	Clinical Suspected Dengue	62	27
31	41	Kaup *et al.*	2014	2013-2014	India	Hospital	Clinical Suspected Dengue	278	62
32	42	Khan *et al.*	2014	2012	India	Hospital	Clinical Suspected Dengue	164	107
33	43	Lall *et al.*	2016	2015	India	Hospital	Clinical Suspected Dengue	3163	646
34	44	Laul *et al.*	2016	2015	India	Hospital	Clinical Suspected Dengue	192	115
35	45	Madan *et al.*	2018	Jun-Aug 2016	India	Hospital	Clinical Suspected Dengue	471	102
36	46	Mehta *et al.*	2014	2008-2011	India	Hospital	WHO	903	253
37	47	Mishra *et al.*	2015	2009-2012	India	Hospital	Clinical Suspected Dengue	433	136
38	48	Mistry *et al.*	2015	2013	India	Hospital	Clinical Suspected Dengue	4366	1802
39	49	Mital *et al.*	2016	2015	India	Hospital	AFI	90	61
40	50	Muruganandham *et al.*	2014	2013	India	Outbreak	WHO	23	13
41	51	Neeraja *et al.*	2014	2011-2013	India	Hospital	Clinical Suspected Dengue	175	109
42	52	Nikam *et al.*	2015	2014	India	Hospital	Clinical Suspected Dengue	1090	300
43	53	Nisarta *et al.*	2016	2015-2016	India	Hospital	Clinical Suspected Dengue	90	21
44	54	Nujum *et al.*	2014	2011	India	Hospital	WHO	851	174
45	55	Padhi *et al.*	2014	2010-2012	India	Hospital	WHO	5102	1074
46	56	Padmapriya *et al.*	2017	2009-2014	India	Hospital	Clinical Suspected Dengue	10099	1927
47	57	Palewar *et al.*	2023	2014-2020	India	Hospital	Clinical Suspected Dengue	6495	4689
48	58	Patankar *et al.*	2014	2012	India	Hospital	Clinical Suspected Dengue	4401	927
49	59	Patil *et al.*	2020	Jan 2019-Dec 2019	India	Hospital	WHO	640	220
50	60	Pothapregada *et al.*	2016	2012-2015	India	Hospital	WHO	398	261
51	61	Prakash *et al.*	2015	2011-2013	India	Hospital	Clinical Suspected Dengue	4019	886
52	62	Prakash *et al.*	2023	2021	India	Hospital	Clinical Suspected Dengue	250	85
53	63	Prudhivi *et al.*	2014	2013	India	Hospital	Clinical Suspected Dengue	1180	284
54	64	Ramachandran *et al.*	2016	2010	India	Hospital	Clinical Suspected Dengue	1666	930
55	65	Rao *et al.*	2016	2013	India	Hospital	Clinical Suspected Dengue	1980	745
56	66	Saravanan *et al.*	2017	2012	India	Outbreak	NVBDCP	600	260
57	67	Saswat *et al.*	2015	2013	India	Hospital	Clinical Suspected Dengue	204	73
58	68	Savargaonkar *et al.*	2018	2012-2015	India	Hospital	Clinical Suspected Dengue	5536	1536
59	69	Shabnum *et al.*	2017	2015	India	Hospital	Clinical Suspected Dengue	1054	456
60	70	Shah *et al.*	2019	2014-2016	India	Hospital	Clinical Suspected Dengue	819	125
61	71	Shaikh *et al.*	2015	2010	India	Hospital	Clinical Suspected Dengue	6554	3202
62	72	Sharma *et al.*	2016	2015	India	Hospital	WHO	60	16
63	73	Sharma *et al.*	2014	2013	India	Hospital	Clinical Suspected Dengue	659	141
64	74	Shobha *et al.*	2014	2013	India	Outbreak	WHO	68	13
65	75	Siddiqui *et al.*	2016	2015	India	Hospital	Clinical Suspected Dengue	7177	2358
66	76	Singh *et al.*	2014	2013	India	Hospital	AFI	1141	812
67	77	Singh *et al.*	2016	2015-2016	India	Hospital	Clinical Suspected Dengue	2709	1538
68	78	Singh *et al.*	2016	2015	India	Hospital	WHO	1100	400
69	79	Singh *et al.*	2023	2022	India	Outbreak	WHO	63280	2060
70	80	Singla *et al.*	2015	2011-2012	India	Hospital	AFI	300	22
71	81	Somasundaram *et al.*	2019	Jun 2017-Nov 2017	India	Hospital	Clinical Suspected Dengue	325	232
72	82	Sushi *et al.*	2014	2011	India	Hospital	AFI	100	8
73	83	Tazeen *et al.*	2017	2014	India	Hospital	Clinical Suspected Dengue	60	48
74	84	Vakrani *et al.*	2017	2013-2015	India	Hospital	WHO	139	101
75	85	Venkatasubramani *et al.*	2015	2010-2012	India	Hospital	Clinical Suspected Dengue	331	49
76	87	Yogeesha *et al.*	2014	2012	India	Hospital	Clinical Suspected Dengue	200	80

**Table 2:** Data set II-DG Age Distribution

**Sr. No.**	**Reference No.**	**Author**	**Year of Publication**	**Year of study**	**Study. Type**	**Avg./Median Age**
1	16	Ahmed *et al.*	2015	2010	Hospital	25
2	89	Athira *et al.*	2018	2015-2017	Hospital	7.6
3	22	Barde *et al.*	2015	2012	Outbreak	33
4	23	Barde *et al.*	2015	2013	Outbreak	35
5	29	Deshkar *et al.*	2017	2012-2016	Hospital	14
6	32	Duthade *et al.*	2015	2014	Hospital	19
7	35	Gusani *et al.*	2017	2014	Hospital	24
8	89	Jain *et al.*	2017	Aug-Nov 2015	Hospital	30.9
9	90	John *et al.*	2019	2014-2018	Hospital	31.3
10	41	Kaup *et al.*	2014	2013-2014	Hospital	26
11	91	Kumar *et al.*	2018	Jan 2013-June 2014	Hospital	7.8
12	48	Mishra *et al.*	2015	2009-2012	Hospital	7
13	92	Mishra *et al.*	2018	2017	Hospital	33
14	49	Mistry *et al.*	2015	2013	Hospital	22
15	56	Padhi *et al.*	2014	2010-2012	Hospital	23
16	58	Palewar *et al.*	2023	2014-2020	Hospital	25
17	59	Patankar *et al.*	2014	2012	Hospital	23
18	60	Patil *et al.*	2020	Jan 2019-Dec 2019	Hospital	35.3
19	93	Pereira *et al.*	2018	Not Mentioned	Hospital	32.41
20	64	Prudhivi *et al.*	2014	2013	Hospital	32
21	66	Rao *et al.*	2016	2013	Hospital	17
22	94	Ravikumar *et al.*	2021	Aug-Dec 2020	Hospital	8
23	67	Saravanan *et al.*	2016	2012	Outbreak	33
24	70	Shabnum *et al.*	2017	2015	Hospital	26
25	95	Sharma *et al.*	2014	2013	Hospital	16
26	83	Sushi *et al.*	2014	2011	Hospital	21
27	96	Swain *et al.*	2019	2010-2016	Hospital	31.6
28	88	Yogeesha *et al.*	2014	2012	Hospital	35
29	97	Esther *et al.*	2023	2012-2017	Hospital	32

**Table 3:** Dataset III-DG Fever (FV) and DG Severity (SV)

**Sr. No.**	**Reference No.**	**Author**	**Year of Publication**	**Year of study**	**WHO Case Definition Reference**	**Dengue Positives**	**DF**	**Severe**
1	12	Abhilash *et al.*	2016	2012-2013	WHO 1997	386	329	57
2	16	Ahmed *et al.*	2015	2010	WHO 1997	1700	1525	175
3	19	Arora *et al.*	2021	2015	WHO 2009	170	106	34
4	89	Athira *et al.*	2018	2015-2017	WHO 2009	34	31	11
5	28	Changal *et al.*	2016	2015	WHO 1997	114	84	30
6	98	Chatterjee *et al.*	2014	2012	WHO 1997	180	128	52
7	99	Chhotala *et al.*	2016	2014-2015	WHO 1997	100	94	6
8	100	Deme *et al.*	2021	August 2018-October 2019	WHO 2012	200	200	116
9	29	Deshkar *et al.*	2017	2012-2016	WHO 1997	3822	3341	481
10	101	Deshmukh *et al.*	2014	2012-2014	WHO 1997	247	173	74
11	30	Dhingra *et al.*	2020	Feb 2014-Oct 2015	WHO 2013	216	94	33
12	90	John *et al.*	2019	April 2014-October 2018	WHO 2012	369	198	171
13	102	Kumar *et al.*	2017	2015-2016	WHO 1997	159		69
14	91	Kumar *et al.*	2018	Jan 2013-June 2014	WHO 2012	40	20	20
15	44	Laul *et al.*	2016	2015	WHO 1997	306	119	56
16	103	Meena *et al.*	2016	2014	WHO 1997	115	89	26
17	104	Mishra *et al.*	2016	2013-2015	WHO 2007	100	84	16
18	105	Misra *et al.*	2015	2003-2014	WHO 1997	97	84	13
19	55	Padhi *et al.*	2014	2010-2012	WHO 1997	116	82	34
20	93	Pereira *et al.*	2018	Not Mentioned	WHO 2009	1074	1048	26
21	106	Pothapregada *et al.*	2015	2012-2014	WHO 2007	550	547	101
22	107	Rathod *et al.*	2018	2013-2015	WHO 2009	254	159	95
23	94	Ravikumar *et al.*	2021	Aug-Dec 2020	WHO 2009	100	100	11
24	108	Sahana *et al.*	2015	2012-2013	WHO 2007	44	43	30
25	73	Sharma *et al.*	2016	2015	WHO 1997	81	61	20
26	109	Sil *et al.*	2016	2015-2016	WHO 1997	16	5	11
27	78	Singh *et al.*	2016	2015	WHO 1997	71	62	9
28	110	Singh *et al.*	2022	Sept-Dec 2019	WHO 1997	400	260	140
29	81	Somasundaram *et al.*	2019	Jun 2017-Nov 2017	WHO 2012	1349	459	34
30	111	Srividhya *et al.*	2017	2013	WHO 1997	232	232	38
31	84	Vakrani *et al.*	2017	2013-2015	WHO 1997	140	70	70

**Table 4:** Dataset IV

**Sr. No.**	**Reference No.**	**Author**	**Year of Publication**	**Year study**	**Total Positive for Dengue**	**No. of Mortality**
1	12	Abhilash *et al.*	2016	2012-2013	386	9
2	112	Acharya *et al.*	2018	2017-2018	364	14
3	15	Ahmad *et al.*	2016	2012-2013	93	4
4	16	Ahmed *et al.*	2015	2010	1700	1
5	21	Barde *et al.*	2014	2011-2012	21	0
6	22	Barde *et al.*	2015	2012	321	5
7	24	Barua *et al.*	2016	2014	101	1
8	113	Bhalla *et al.*	2014	2011	299	2
9	25	Bhattacharya *et al.*	2017	2013	168	0
10	98	Chatterjee *et al.*	2014	2012	180	7
11	99	Chhotala *et al.*	2016	2014-2015	100	4
12	29	Deshkar *et al.*	2017	2012-2016	3822	40
13	101	Deshmukh *et al.*	2014	2012-2014	247	11
14	114	Deshwal *et al.*	2015	2013	515	4
15	30	Dhingra *et al.*	2020	Feb 2014-Oct 2015	216	13
16	32	Duthade *et al.*	2015	2014	233	5
17	90	Jain *et al.*	2017	2015	369	19
18	115	Krishnamoorthy *et al.*	2017	2013	1308	23
19	105	Mishra *et al.*	2016	2013-2015	97	1
20	116	Nagaram *et al.*	2017	2015-2016	174	9
21	51	Neeraja *et al.*	2014	2011-2013	109	9
22	117	Nimmagadda *et al.*	2014	2010-2012	150	3
23	118	Padyana *et al.*	2019	2015	1170	20
24	119	Pai Jakribettu *et al.*	2015	2013-2014	60	2
25	106	Pothapregada *et al.*	2015	2012-2014	254	6
26	106	Pothapregada *et al.*	2015	2012-2014	261	6
27	62	Prakash P	2023	2021	85	2
28	65	Rao *et al.*	2016	2013	745	0
29	108	Sahana *et al.*	2015	2012-2013	81	2
30	120	Sahu *et al.*	2014	2011-2013	486	5
31	66	Saravanan *et al.*	2016	2012	260	7
32	121	Saroch *et al.*	2017	2015	172	16
33	72	Sharma *et al.*	2016	2015-2016	200	0
34	73	Sharma *et al.*	2016	2015-2016	107	0
35	95	Sharma *et al.*	2014	2013	141	0
36	76	Singh *et al.*	2014	2013	812	12
37	79	Singh *et al.*	2023	Sept-Dec 2019	1349	6
38	122	Singhal *et al.*	2020	2017	575	15
39	111	Srividya *et al.*	2017	2013	140	1
40	84	Vakrani *et al.*	2017	2013-2015	101	0

**Table 5:** Dataset V

**Sr. No.**	**Reference No.**	**Author**	**Year of Publication**	**Year of study**	**Total Tested**	**Primary (PM)**	**Secondary (SC)**
1	22	Barde *et al.*	2015	2012	115	111	4
2	28	Changal *et al.*	2016	2015	114	38	76
3	33	Gopal *et al.*	2016	2013	25	13	12
4	41	Kaup *et al.*	2014	2013-2014	62	52	10
5	42	Khan *et al.*	2014	2012	87	82	5
6	104	Mishra *et al.*	2016	2013-2015	94	83	11
8	51	Neeraja *et al.*	2014	2011-2013	109	87	22
9	52	Nikam *et al.*	2015	2014	300	224	76
10	56	Padmapriya *et al.*	2017	2009-2014	1752	1124	628
11	65	Rao *et al.*	2016	2013	22	21	1
12	123	Rashmi *et al.*	2015	2014	97	93	4
13	114	Shabnum *et al.*	2017	2015	456	442	14
14	75	Siddiqui *et al.*	2016	2015	76	24	52
15	84	Vikram *et al.*	2016	2013	22	8	14

**Table 6:** Dataset VI

**Sr. No.**	**Reference No.**	**Author**	**Year of Publication**	**Year study**	**Total Tested**	**Tested as Seropositive**
1	125	Alagarasu *et al.*	2023	2009-2019	2451	1963
2	20	Badoni *et al.*	2023	2018-2019	279	143
3	126	Garg *et al.*	2017	2011-2012	2558	1525
4	127	Lakshmi *et al.*	2022	2016-2019	5147	1314
5	124	Mishra *et al.*	2018	2017	1434	1163
6	128	Murhekar *et al.*	2019	2017-2018	12300	5338
7	129	Oruganti *et al.*	2014	Not mentioned	200	179
8	59	Patil *et al.*	2020	Jan 2019-Dec 2020	640	398
9	130	Rodríguez-Barraquer *et al.*	2015	2011	800	744
10	131	Vikram *et al.*	2016	2013	1899	542
